# Research consultation clinic: impetus towards facilitating primary care research

**DOI:** 10.1186/1447-056X-12-4

**Published:** 2013-09-16

**Authors:** Ngiap Chuan Tan, Yang Thong Tan, Patricia T Kin

**Affiliations:** 1 Research Department, SingHealth Polyclinics Head Office, 167 Jalan Bukit Merah, Connection One, Tower 5, #15-10, Singapore 150167, Singapore; 2 DUKE-NUS Graduate Medical School, 8 College Rd, Singapore 169857, Singapore

**Keywords:** Research, Consultation, Primary care

## Abstract

**Background:**

In Singapore, SingHealth Polyclinics (SHP) is an accredited Family Medicine (FM) training centre which managed 1.8 million primary care patient-visits in 2012. To promote research in the institution, research consultation clinics (RCC) are being introduced in 2010 to enable free face-to-face consultation between experienced and novice researchers on specific research topics. Each RCC session allows about an hour or more for the SHP staff, medical undergraduates and general practitioners to seek advice and clarification on key research areas, ranging from research question refinement, study design and execution, data analysis, result presentation to publication. The consultants comprise of two FM researchers with postgraduate research qualification.

**Aim:**

This article aims to review the implementation of RCC from 2010 to 2012 and its impact on research activities and outcome indicators in the same period of time.

**Methods:**

The study comprised of two segments. Part I was a three-year retrospective review of the RCC administrative record. The total number of RCC sessions, hours utilised, participants’ profiles, the number of research studies initiated by them and their research presentations at local and overseas scientific meetings/conferences were computed. Part 2 was an anonymous web-based questionnaire survey fielded to RCC participants to collect their feedback on the RCC service and their self-reported initiation and completion of research study after the RCC consultation.

**Results:**

The RCC sessions increased from 17 to 40 sessions, resulting in increment of 2 to 14 research presentations and from 2 to 6 initiations of new research studies per annum from 2010–2012. The response rate to the questionnaire survey was 70.3%, with the majority of multi-disciplinary respondents rated the RCC service to be accessible, adequate and were satisfied with its quality. Study design, data management and study execution were ranked as important areas of research for consultation. 79% of them had started a research project and 36% had completed their studies.

**Conclusions:**

The RCC is a feasible model to catalyse multi-disciplinary research in primary care institutions. Further study is needed to evaluate its relevance when research advances and novice researchers become experienced investigators to take on more complex projects.

## Background

A strong primary healthcare system is vital to improve the health of the general population and reduce the healthcare burden to the nation [1,2]. Research in primary care aims largely to seek better understanding of disease management in relation to the individuals, families and the community, and to evaluate the effectiveness and efficiency of healthcare practices and health policies [3]. Beyond general practice and family medicine, primary care research is multi-disciplinary, covering community nursing and pharmacy, sociology, health service and policy research. As primary care supports the base of the population pyramid and amidst a rapidly expanding global population, there is an urgency to scale up primary care research to seek medical evidence to prevent, cure, and care for diseases; to translate these evidences into regular clinical practice and to formulate policies to ensure universal equity of care [4].

However, primary care research faces challenges, even in resource-rich countries, due to limitations of research capacity in general practice, inadequate research infrastructure in primary care and poor provision of support to individual general practitioners to conduct research. Saad H. Al-Abdullateef in a survey of general practitioners in selected primary care centres in Saudi Arabia showed that while a majority expressed interest (66%) and planned to carry out research (74.2%), they cited insufficient time (83.5%) and lack of support (58.8%) as key barriers to research [5].

Practice-based research networks (PBRNs) are groups of practices networked together to undertake research relevant to general practice and the local community's needs [6,7]. These PBRNs, often involving collaborations between practitioners and academicians, can expand the capacity and bandwidth of primary care physicians [8]. Nonetheless, these PBRNs are largely medical, whist nurses and other researchers tend to form their respective research networks. Furthermore, a survey of a New York based PBRNs revealed that only 25% of the physicians had formal research training and 21% of them had clinical research certification [9]. Lack of time or competing demands for time was the top ranked barrier (92%) in the survey, and 80% of the clinicians reported that their lack of appropriate training was a barrier [9]. Thus, participation in PBRNs may not adequately address the specific research needs nor provide training for the individual primary care researchers; neither does it add impetus towards multi-disciplinary research in primary care.

Nevertheless, there are ample research related training opportunities that are available from the local academic institutions to equip primary care professionals with basic research skills. Basic research training is incorporated in medical undergraduate education curriculum, and in the respective master degree programmes for community nurses and pharmacists who take on advanced professional training.

Aside from training, mentorship appears to be pivotal in moulding primary care practitioners into researchers. Curtis P et al. reported that amongst Family Medicine (FM) graduates who received the American National Research Service Awards, they were less successful in terms of research and publication output compared to fellow recipients such as internists and paediatricians [10]. The authors attributed the differences to a lack of protected time and sustained research mentorship for the family physicians. Likewise, in Israel, FM residents trained under pro-research mentors were likely to be more intensely involved in research when they became family physicians [11].

A strong and increased level of commitment from primary care organisations is needed to provide support and resources and to reduce barriers for family physicians to carry out research [12]. Hannaford P et al. showed that a modest investment of resources and support will create substantial increases in both the quality and quantity of primary care research [13]. They recommended that this investment should be targeted at both existing primary care professionals working in service settings in primary care and those in the academic departments in the universities [13]. Various funding models for primary care research have since been developed and implemented in various countries, centred largely on channelling government funding via FM departments in universities in Australia & the Netherlands, or via the Royal College of General Practitioners in Britain [14-16]. However how effective and efficient are these funding models in promoting primary care research and enhance research-related training for the general practitioners are seldom measured, and its translation into actual research endeavours are rarely evaluated. A new model to expedite primary care research in terms of effective individual coaching and efficient application is urgently needed to empower the primary care practitioners.

Primary care research faces similar challenges in Singapore, where infrastructure constraints and healthcare resources were hithertho barriers to its development [17]. However in recent years, despite the lack of dedicated research funding for primary care researchers from the local official research agencies, rapid development of academic medicine in the local public healthcare institutions resulted in unprecedented support for primary care research from primary healthcare organisations, such as SingHealth Polyclinics (SHP). The latter comprises a cluster of nine public primary care clinics or polyclinics in Singapore, which is also a recognised FM training centre accredited by the American College of General Medical Education. Whilst governance, policy and finance are centrally administered in SHP, the polyclinics operate at various sites to serve the local communities in the southern and eastern parts of Singapore using a common and integrated electronic medical records system. Thus in terms of primary care research, the institution can be regarded as a de-facto practice-based multi-disciplinary research network.

Novice researchers from SHP can seek seed funding from SHP to support their approved research projects whilst established researchers are encouraged to vie for competitive research grants from national research agencies. Whilst financial support to kick-start research for these researchers is no longer a barrier, approval for seed funding is based on the evaluation of research proposals’ scientific merits and logistic considerations. Hence novice researchers find it propitious to consult experienced researchers for guidance in order to optimise their success rates in their seed funding or other grant applications.

With the endorsement from senior management in SHP, research consultation clinics (RCC) are established in May 2010 to support budding FM researchers in the institution. Dedicated resources are channelled centrally from institution annual operating budget to set up the RCC service. This caters to the opportunistic cost for the consultants, as well as the participants to meet at the RCC for free face-to-face consultations during clinic operating hours. It also provides for the seed funding for researchers to support the execution of their project.

Each RCC session allows at least an hour for any individual or group of multi-disciplinary SHP staff to seek advice and clarification on any issues pertaining to their research endeavours. During the consultation sessions, the topics covered include the various domains in research, ranging from exploring research idea, definition of research question, research method, project design, application for ethics committee review, logistic planning, data analysis and interpretation to results presentation and publication. The participants can arrange for multiple consultations to clarify queries for their same study or repeat consultation for another study.

The RCC sessions are taken up by SHP multi-disciplinary staff, including doctors, nurses and pharmacists who plan to commence research as part of their professional development or are pursuing advanced training programmes which incorporate research as part of the curriculum. The service is also open to external primary care researchers, including private general practitioners, and medical students from the two local medical colleges, under the over-arching objective of enhancing primary care research development and foster research collaborations between different primary healthcare providers in Singapore. This necessitates the increase of consultants from one to two to manage the expanded RCC capacity. The consultants are FM researchers within the institution with post-graduate qualifications in research.

Periodic evaluation of the service provided by RCC from the participants is vital to ensure that this model of research support remains relevant and its service caters adequately to their needs. This study aims to determine the level of satisfaction amongst the RCC participants in terms of their perception of the accessibility of the service, adequacy of the duration of the consultation, effectiveness of the consultancy, the key research areas in which they seek consultation, and the outcomes of the RCC consultation.

## Methods

The evaluation of the RCC service comprises of two segments. The first part was a retrospective review of the RCC records from 2010–2012 to determine the short-term accomplishments of past participants in terms of their research study initiation and subsequent oral or poster presentation at academic meetings and/or scientific conferences. “New study” is defined as study initiated within the respective calendar year with reference to its date of approval from the institutional review board. The research presentations refer to investigators’ presentations of their research findings at local or overseas scientific meetings or conferences which are approved by SHP. Both outcomes were traced from the respective databases in the SHP Department of Research.

The second segment was a questionnaire survey of the same cohort of participants. Each participant was invited to undertake a web-based questionnaire survey regardless of the frequency of RCC participation. The sampling frame comprised of participants who had utilised the RCC service at least once and who had provided their email contact. The records show a list of 75 RCC participants between January 2010 and December 2012, of which email addresses were provided by 64 of them.

The questionnaire collected data on the participant’s

1. demographic profile such as age, gender, education status, staff category

2. views on accessibility of the service,

3. quality of the consultancy,

4. ranking of key research domains for which they sought consultation

5. outcome measures of RCC in terms of initiation and completion of their respective research projects

The questionnaire survey was anonymous to ensure truthful responses from the participants. The investigators notified the participants of the survey via their email addresses. Second and third round of notices were delivered to the participants’ email addresses to serve as reminders to participate in the survey on the second and third week respectively after the commencement of the first survey.

The survey was designed using the web-based Qualtrics platform to field the questionnaire. The participants would read the study’s objectives and other research-related information according to the Participant Information Sheet prior to answering the questionnaire. Due to anonymity of the questionnaire and the minimal risk nature of the study, approval was obtained from the Institutional Review Board (CIRB reference no: 2013/253/E ) to waive the written consent of the participant. The data from the completed questionnaire were imported into MS Excel for computation and analysis.

## Results

**Figure 1:**
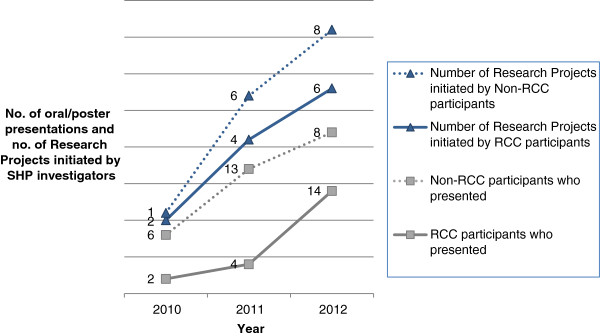
New research projects initiated in SHP and research presentation by SHP staff.

The number of RCC sessions and hours increased from 17 sessions (17 hours) in 2010, 47 sessions (48.5 hours) in 2011, to 40 sessions (46 hours) in 2012. The number of new research projects or conference presentations resulting directly from the investigators’ participations in the RCC also increased from 2010 to 2012 (Figure 1).

For the questionnaire survey, 64 email addresses from a list of 75 participants were found to be unique and currently valid. 45 of them responded to the survey, constituting a response rate of 70.3% (45/64). 39 of them completed the entire questionnaire, which formed the denominator for the computation of the results.

Majority of participants were medical staff of SHP (56%), predominantly females (67%), aged 21–40 years (58%) and with post-graduate qualifications (49%). Table 1 shows the demographic profile of participants in the questionnaire survey.

**Table 1:** Demographic profile of RCC participants

	**Retrospective review of department record**		**Questionnaire survey**	
**Demographic characteristics**	**Number (n) Total = 64**	**%**	**Number (n) Total = 45**	**%**
Domain of work				
SHP Staff	43	67	33	73
Non-SHP Staff (e.g. GP, Medical students)	21	33	12	27
Gender				
Male	23	36	15	33
Female	41	64	30	67
Age group (Year)				
21-40	37	58	26	58
41-60	26	41	18	40
61 and above	1	2	1	2
Education status				
Undergraduate	12	19	6	13
Degree holder	21	33	17	38
Post graduate	31	48	22	49
Profession				
Medical (Medical students and GP inclusive)	39	61	25	56
Nursing and pharmacy	25	39	20	44

**Figure 2:**
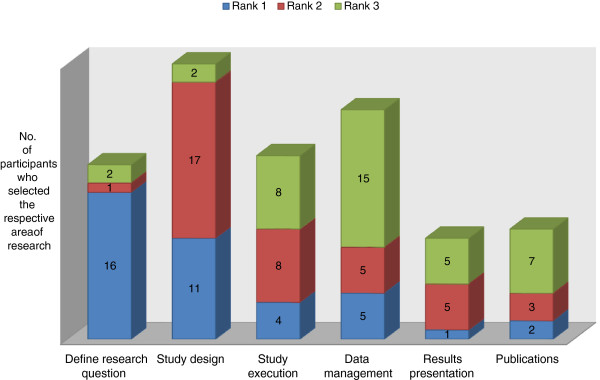
Participants’ ranking of top three key areas of research for their consultation at RCC.

**Figure 3:**
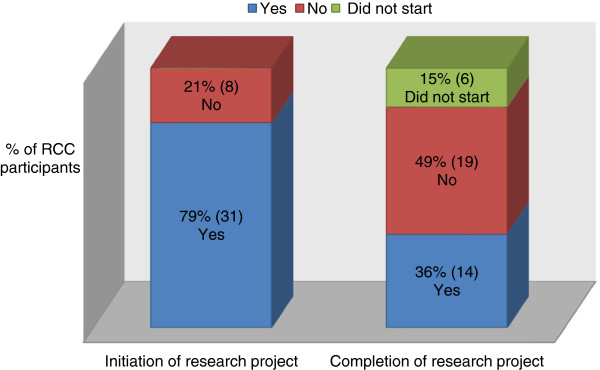
Outcomes of consultation at RCC.

Most participants considered the RCC as accessible and adequate in time provision. All of them were satisfied with the quality of consultancy at RCC (Table 2). Study design, data management and study execution were ranked as the key areas of research for which participants sought consultation at RCC (Figure 2). Almost 8 out of 10 (79%) participants had started a research project after RCC consultation and 36% (14/39) of them had completed their studies (Figure 3).

**Table 2:** Accessibility, quality and perceived value of consultancy at RCC

**Accessibility to RCC**		
**Views of participants n = 39**	**Total disagree/disagree n/(%)**	**Totally agree/agree n/(%)**
I can easily book a RCC appointment.	4 (10)	35 (90)
I feel that the RCC, currently held on two afternoons per week are adequate.	7 (18)	32 (82)
The venue at a centralised location in HQ is convenient.	1 (3)	38 (97)
The one-hour time slot per RCC session is adequate.	8 (21)	31 (79)
**Quality and perceived value of consultancy at RCC**		
**Views of participants n = 39**	**Agree n/(%)**	**Totally agree n/(%)**
The consultant at RCC is qualified to provide the consultation.	21 (54)	18 (46)
The consultation provides the information that I need for my research project.	23 (59)	16 (41)
The consultation helps in my research.	22 (56)	17 (44)
I appreciate that RCC service is currently free of charge.	12 (31)	27 (69)

Some investigators have already started their study and sought consultation at RCC in the midst of their study execution. This accounted for the difference between those who indicated that they had initiated their research (79%) and those who have completed or in the process of carrying out their studies (36% + 49% or 85%).

## Discussion

The study shows that the establishment of RCC can be a catalyst to facilitate research in primary healthcare institution. It is a system approach to empower novice researchers to embark on research. The RCC serves as a platform for researcher-centric coaching, which caters to their individual or team’s specific needs when they plan or are in the midst of executing research. Most of the participants have undertaken basic research training at local academic institutions. However these generic structured training programs may not provide answers to specific questions or solutions to problems encountered by the individual or team members during the planning or execution stages. The RCC bridges this gap, with the consultants functioning as mentors to guide these researchers. Once these researchers gain hands-on experience and confidence after these beginners’ trials or studies, it sets the stage for them to pursue advanced research training program and to take on more complex studies.

The key elements for a successful set-up include support from senior management to provide the appropriate resources, ensuring that the service is accessible and convenient. Adequate time allocation and quality of the consultancy are essential elements for successful service delivery to the participants. Based on their rankings, most participants value the consultation in critical areas of research, such as study design, execution and data management. These components will appeal to novice researchers who are commencing their early phases of research planning and execution. Nonetheless, established researchers can also use the RCC service, as peer reviews by consultants serve to polish up their presentations and publications.

This study is the first in the healthcare institution and provides a timely evaluation of the needs of the staff members, who are planning or have already embarked on research for the past three years. However, about one in five participants have yet to initiate their study after the RCC consultation. Further research will be required to understand their other unmet needs and identify other barriers that could have hindered their initiation. There is also a lag time between research consultation and the initiation of new studies due to logistic reasons such as application for ethics approval and lack of research funding.

The evaluation of the research support service needs to be implemented on a regular basis to stay relevant to the evolving research developments in SHP. As novice researchers become more experienced and take on more complex research studies, the service will need to expand. To enhance the RCC service, additional expertise is required to augment the small pool of family physicians cum consultants, including biostatistician, epidemiologist and bioinformatics professional. A biostatistical consultation clinic, facilitated by a biostatistician, has been set up in SHP to complement the RCC in September 2012. Further study will be carried out to determine its effectiveness in providing biostatistical support to the researchers. Gathering qualitative feedback from the consultants on the RCC service will close the evaluation loop in future study.

One limitation of the survey is the failure of the investigators to reach out to all the RCC participants. These include individuals who had left the organisation or medical students who had already graduated, resulting in change of their email addresses. Administrative support will need to be enhanced to ensure that the participants’ contacts are periodically updated, which may ameliorate the response rate of subsequent surveys. In addition, absolute and opportunistic cost of providing the resources in operating the RCC, including time and manpower, will be computed in future surveys, as the basis of cost effectiveness analysis.

## Conclusions

The establishment of research consultation clinic in a primary healthcare institution helps to support and facilitate research amongst its staff. Accessibility, convenience, adequacy and quality of the consultancy are key elements for its successful implementation. It can be measured by process outcomes such as the number of new research projects initiated and publications in journals.

## Competing interests

The authors declare that they have no competing interests.

## Authors’ contributions

TNC contributed in the conception and design of the study, the content of the survey questions, analysis of data publication and manuscript drafting. KPT contributed in the content of the survey questions, survey distribution and the review of manuscript. TYT participated creating the web base survey, collection and processing of data and the review of manuscript. All authors read and approved the final manuscript.

## Authors’ information

KPT is the manager of department of research, SingHealth Polyclinics. TYT is the executive of department of research, SingHealth Polyclinics. TNC is the director of department of research, SingHealth Polyclinics. He is also a family physician (senior consultant) at SingHealth Polyclinics (Pasir Ris), an adjunct assistant professor of Duke-NUS Graduate Medical School.
